# Multidimensional needs of patients living and dying with heart failure in Kenya: a serial interview study

**DOI:** 10.1186/s12904-018-0284-6

**Published:** 2018-02-17

**Authors:** Kellen N. Kimani, Scott A. Murray, Liz Grant

**Affiliations:** 1 School of Public Health, College of Health Sciences, University of Nairobi, P.O. Box 30197, GPO, Nairobi, Kenya; 2 Primary Palliative Care Research Group, the Usher Institute, University of Edinburgh, Edinburgh, EH8 9AG UK; 3 Global Health Academy and the Primary Palliative Care Research Group, the Usher Institute, University of Edinburgh, Edinburgh, EH8 9AG UK

**Keywords:** Heart failure, Patient experience, Palliative care, Qualitative, Serial interviews, Kenya, Sub Saharan Africa

## Abstract

**Background:**

Heart failure is an emerging challenge for Sub Saharan Africa. However, research on patients’ needs and experiences of care is scarce with little evidence available to support and develop services. We aimed to explore the experiences of patients living and dying with heart failure in Kenya.

**Methods:**

We purposively recruited 18 patients admitted with advanced heart failure at a rural district hospital in Kenya. We conducted serial in depth interviews with patients at 0, 3 and 6 months after recruitment, and conducted bereavement interviews with carers. Interviews were recorded, transcribed into English and analyzed using a thematic approach, assisted by Nvivo software package.

**Results:**

Forty-four interviews were conducted. Patients experienced physical, psychosocial, spiritual and financial distress. They also had unmet needs for information about their illness, how it would affect them and how they could get better. Patients experience of and their interpretation of symptoms influenced health care seeking. Patients with acute symptoms sought care earlier than those with more gradual symptoms which tended to be normalised as part of daily life or assumed to be linked to common treatable conditions. Nearly all patients expected to be cured and were frustrated by a progressive illness poorly responsive to treatment. Accumulating costs was a barrier to continuity of care and caused tensions in social relationships. Patients valued information on the nature of their illness, prognosis, self-care, lifestyle changes and prevention strategies, but this was rarely available.

**Conclusions:**

This is the first in-depth study to explore the experiences of people living with advanced heart failure in Kenya. This study suggests that patients would benefit from holistic care, such as a palliative approach that is aimed at providing multidimensional symptom management. A palliative approach to services should be provided alongside chronic disease management aimed at primary prevention of risk factors, and early identification and initiation of disease modifying therapy. Further research is needed to determine best practice for integrating palliative care for people living and dying with heart failure.

## Background

Heart failure is a major cause of global cardiovascular morbidity and mortality. About 26 million people are living with heart failure worldwide and this burden is increasing [1]. Population based surveys conducted in North America, Europe, Australia and parts of Asia have found that 1–2% of people have heart failure [2]. While similar estimates are not currently available for Sub Saharan Africa (SSA), findings from available data from hospital based studies show that heart failure is the most common primary diagnosis of patients admitted with cardiovascular disease and accounts for up to 9–15% of hospital admissions [3, 4].

Despite advances in medical therapy heart failure prognosis remains poor. Globally, 30–40% of patients die within a year of diagnosis and majority within five years [2, 5]. Poorer survival rates are reported in SSA with 6-month morality rates nearing 20% [6]. As SSA experiences expanding urbanisation, improved communicable disease control with better survival into adulthood and changes in nutrition and lifestyles, the burden of heart failure is likely to increase. By 2030, non-communicable diseases are estimated to overtake infectious diseases as the leading cause of death in the sub-continent [7, 8].

A double burden of non-communicable and communicable diseases presents significant challenges for designing health services capable of responding to emerging population needs. Infectious and maternal and child health conditions remain significant health priorities for SSA and current services structured for mainly acute episodic care may be unprepared to cater to the dynamic needs of patients with chronic illness including those with heart failure [9, 10]. The heart failure trajectory is complex and unpredictable, with patients experiencing progressive physical decline interspersed with acute exacerbations which are potentially fatal [11, 12]. Findings from qualitative studies mainly conducted in economically developed countries show that in addition to the burden of physical symptoms, patients with heart failure experience psychological, spiritual and social distress with poor understanding of their illness, treatment and prognosis [13–15].

We set out to explore the experience of patients living with advanced heart failure in Kenya. We wanted to understand from patients, in their own words, what it is like for them to live with their illness. This type of research supports patients to share their experiences and become active participants in suggesting how services can be better improved to help them. Until recently, most of what we know about the experience of living with heart failure is from qualitative studies conducted in economically developed countries [16]. Research evidence is now emerging from SSA with findings published from a serial interview study conducted in Uganda [17]. However, more evidence is needed from SSA to represent the rich ethnic diversity and account for differences in social, cultural and health systems contexts.

## Methods

### Aim

We aimed to understand how patients made sense of their illness as well as examine how their social and cultural context may have influenced their interpretations using a constructivist epistemological approach [18, 19].

### Design

We employed a longitudinal qualitative study design using serial in-depth interviews to explore patients’ multi-dimensional experiences. Longitudinal methods are useful for capturing patients’ changing needs and preferences along the course of illness. These methods are appropriate for conducting sensitive research as they allow time for trusting relationships to develop between the researcher and participants. Trusting relationships enable the exploration of difficult topics which might not be possible with single interviews [20]. These methods have been used to explore the experiences of people living with chronic illnesses such as cancer, advanced chronic obstructive pulmonary disease and multiple chronic illnesses [21–23].

### Setting

We purposively identified 53 patients admitted with advanced heart failure at a district hospital in a predominantly rural area in central Kenya. We approached patients who were breathless with less than ordinary physical activity or breathless at rest (New York Heart Association Grade III or IV) [24], and those reflecting the epidemiology and sociodemographic characteristics of heart failure in this setting. Patients with cognitive impairment, a more advanced other life-threatening illness requiring most care, and living 35 km beyond the district hospital were excluded.

### Participant recruitment

We requested hospital clinical staff to identify potential patient participants fitting the inclusion criteria, to provide them with information about the study and confirm their willingness to participate. Potential patient participants were also made aware that they could chose a carer to be interviewed when they became too unwell to take part in the study. We anticipated that some patients would wish not to select a carer, and in such instances, we respected their preference.

### Data generation

Patients who consented were interviewed by KNK, a Kenyan female public health doctor who, could speak Kiswahili and English. Face-to-face interviews with patients were carried out at three monthly intervals up to a maximum of 6 months. However, interview timings were flexible and dependent on a patient’s health status or the occurrence of an event, such as a hospital admission, triggering an earlier or later interview. Interviews took place in hospital wards or at a patient’s home, and lasted 25–125 min. All interviews were audio recorded and transcribed into English. A topic guide was used to direct interviews and explore patients’ physical, social, spiritual experiences including their experiences with care. We explored similar themes with carers. Patients were phoned monthly to record changes in their condition, to plan subsequent interviews and to maintain contact. Field notes were used to record observations made.

Ethical approval was granted by Kenyatta National Hospital – University of Nairobi ethics and research committee in Kenya and Centre for Population Health Sciences ethics committee at the University of Edinburgh. Written and signed or inked consent was obtained from participants at the start of the first interview and confirmed verbally in subsequent interviews.

### Data analysis

We were guided by Braun and Clark’s [25] multi staged approach to thematic analysis. In the initial data familiarization phase, transcripts were read repeatedly to understand the width and breadth of the data. Codes were then identified and arranged into themes and sub-themes illustrating patients’ experiences. Themes were regularly reviewed to ensure data were coherent and variations between themes were distinguishable. Multidisciplinary team meetings were held regularly with experienced palliative care researchers (LG, SAM) to consider emerging codes, themes and data saturation. Nvivo qualitative data analysis package was used to manage data.

## Results

We identified 53 patients of whom 18 (10 female; 8 male) agreed to take part, 22 declined, and 11 were found unsuitable for inclusion on further review. Eighteen carers were chosen for interviewing. Three patients died and four were deemed untraceable by the end of the study. Thirteen participants took part in the second set of interviews and 11 participants in the third set of interviews. Of the eighteen carers invited for interviewing, two bereavement interviews were held. Table 1 summaries study participants. This paper provides an in depth account of the broader dimensions of patients’ experiences and supplements previously reported data on the spiritual issues of participants published in 2016 [26]. We found that patients experienced multidimensional problems including physical, psychological, social and financial problems. They also had little access to information on their illness including prognosis and treatment.

**Table 1:** Characteristics of patients recruited for this study

Participant No	Aetiology of heart failure	NYHA class	Duration in study (months)	Alive or Dead at end of study
1.	Hypertensive heart disease	III	6	Alive
2.	Hypertensive heart disease	IV	6	Alive
3.	Dilated cardiomyopathy	IV	6	Alive
4.	Cor pulmonale	III	6	Alive
5.	Cor pulmonale	IV	6	Alive
6.	Hypertensive heart disease	IV	2	Dead^b^
7.	Dilated cardiomyopathy	III	6	Alive
8.	Hypertensive heart disease	III	6	Alive
9.	Hypertensive heart disease	III	1^a^	Not known
10.	Cor pulmonale	IV	5	Dead^c^
11.	Hypertensive heart disease	III	6	Alive
12.	Unknown	IV	6	Alive
13.	Hypertensive heart disease	III	6	Alive
14.	Hypertensive heart disease	IV	1	Dead^b^
15.	Dilated cardiomyopathy	III	1^a^	Not known
16.	Hypertensive heart disease	III	6	Alive
17.	Hypertensive heart disease	III	5^a^	Not known
18.	Cor pulmonale	IV	1^a^	Not known

*NYHA* New York Heart Association, ^a^deemed untraceable at end of study period, ^b^bereavement interviews held, ^c^carer untraceable

### Physical symptoms

The onset of illness was characterized by multiple physical symptoms. Breathlessness, fatigue, ankle oedema and cough were frequently reported. In particular, acute onset breathlessness caused significant anxiety and triggered a decision to seek emergency care.

It all started with breathlessness. I felt like I was having an asthmatic attack. It was hard to breathe and I knew I was in danger…. I needed a hospital. *[Participant 7, 45–54 years, 1st interview, dilated cardiomyopathy (DCM)].*

Conversely, gradual onset breathlessness went unnoticed and was often placed in the context of patients’ daily lives. This led patients to seek care when their illness was at an advanced stage.

I was breathless for many years. I thought it was because of walking up the hill on my way home. *(Participant 10, 45–54 years, 1st interview right sided heart failure’ [RHF) from chronic obstructive pulmonary disease (COPD)].*

Others described their condition as ‘*ugonjwa ya kawaida’* – a mild and ordinary illness - in reference to common acute and treatable illnesses prevalent in this context. This often delayed appropriate care as patients self-treated before they decided to see a health professional.

It started like a normal illness. I was breathless, tired and did not want to do much. It was just like malaria or pneumonia. *(Participant 18, 30–39 years, 1st interview, RHF with COPD).*

Once medical treatment was started and symptoms abated, some patients misunderstood this to mean they were healed. Patients were hopeful that the illness was curable and symptoms would not recur.

I am better now. It’s not like before…. I don’t think I have a heart problem anymore. *[(Participant 8, 45–54 years, 2nd interview, hypertensive heart disease(HHD)].*

However, those with poorly controlled symptoms expressed frustration of living with a progressive illness leading to frequent hospital visits and admissions. A few questioned the nature of their illness with some even expressing a preference for a HIV diagnosis.

This illness is not good…. why did I get it? It’s worse compared to HIV. At least there is treatment for HIV and you will feel better. Now I get tired easily and my heart beats so fast…I am afraid I will collapse any time. *(Participant 15, 25–34 years, 1st interview, DCM).*

### Psychological issues

Although most were anxious about their illness, few openly expressed worry. Most patients responded stoically accepting their illness as fate or as God’s will.

When things have gone wrong they have gone wrong. What can you do? Even if I think about it...I won’t get better. I would rather accept my life as is and pray to God to help me. *(Participant 13,55–64 years, 2nd interview, HHD/diabetes).*

Younger patients experienced significant anxiety and depression. They expressed concerns about not getting better which prevented them from getting back to work to support their families and pay for their care. Worsening symptoms led to hopelessness.

I feel terrible when my body starts swells again…. I think that I am going to die*. (Participant 16, 25–34 years, 2nd interview, HHD).*

However, patients whose symptoms responded to treatment expressed positive emotions with expectation for a cure.

I am hoping to get better. When I go for my last clinic appointment I think they will give me a clean bill of health (*[Participant 7, 45–54 years, 2nd interview, DCM).*

### Social issues

Family, friends, church members and co-workers offered social support. Social networks offered a sense of community where patients could share their experiences and receive encouragement to ease their distress.

My friends come to visit. They support me to remain positive. They don’t want me to worry about my illness or about the future. *(Participant 8, 45–54 years, 2nd interview, HHD).*

Although social networks were helpful, the chronic nature of the illness challenged relationships. Several patients described how family and friends withdrew their support as the illness progressed. Financial help was of utmost importance as patients were not physically able to work.

Mum was not doing too well so she asked her brother to come see her. It’s like she knew she was going to die. He only came once and never returned*. (Bereavement interview with carer of participant 6, 55–64 years).*

Before this illness my father and I had a good relationship. After I fell ill he left us. He did not have money to pay the hospital bill. (*Participant 2, 35–44 years, 1st interview HHD/diabetes/HIV).*

Some patients experienced stigma and struggled to establish new relationships.

I want to marry and start a family but when I tell someone about my illness they feel like they don’t have a future with me. They are afraid that children can inherit this illness. *(Participant 7, 45–54 years, 1st interview, DCM).*

### Financial issues

Many patients were caught off guard by the financial costs of long term care. Some described how accumulating costs created an endless cycle of poverty, which in turn created a further cycle of illness.

Being ill means being poor. If you are ill you can’t work and if you can’t work you can’t afford to the medicines you need. *(Participant 6, 55–64 years, 1st interview, HHD/myocardial infarction/asthma).*

High health care costs limited the financial resources available to meet other household needs such as rent and school fees.

Those drugs cost a lot and I also need to pay school fees. The household budget is not enough for this. After paying the fees, I only have enough money to buy medicine for 2 weeks. *(Participant 8, 45–54 years, 1st interview, HHD).*

Health care expenses included indirect costs such as time off work, domestic assistance and transportation. Patients not able to meet transportation costs missed hospital appointments which interrupted continuity of care.

The doctor told me to go back to the hospital for a clinic appointment but the consultation fee was expensive and I couldn’t afford it. Also, I did not have enough money to get to the hospital… so I skipped my appointment and went to a nearby pharmacy and bought the drugs I needed. *(Participant 10, 45–54 years, 1st interview, RHF with COPD).*

When the cost of care became prohibitive patients relied on family and friends for financial help. A few who were in formal employment and were compulsory members of the national social health insurance scheme received help with paying for care.

The national health insurance is good because I do not have to pay for anything at the hospital…even lab tests are covered. *(Participant 1, 55–64 years, 3rd interview, HHD/diabetes).*

### Information needs

At diagnosis, many patients were anxious as they were not aware of an illness affecting the heart.

I was shocked by the diagnosis. After the doctor spoke to me I felt worse. I had never heard of a disease affecting the heart. *(*Participant 8, 45–54 years, 1st interview, HHD).

Most were told by health providers they had a ‘heart problem’ and few understood what this diagnosis meant. Health providers used metaphors such as a swollen heart to capture the illness but this exacerbated anxiety. Patients were concerned about the physical structure of their heart and what would happen in the future.

I was told that my heart is swollen. I was afraid it would burst and I could die anytime. *(Participant 16, 25–34 years, 2nd interview, HHD).*

Although patients valued information on how they could prevent their illness reoccurring, this information was not forthcoming. This was particularly challenging as most patients understood their illness as an acute condition which could be treated and prevented, such as a common illness like malaria.

I want to know what I can do so that this does not happen again. That would be helpful. (*Participant 3, 65–74 years, 1st interview, DCM).*

Nearly all patients expected a cure and understood their condition as a short-term illness. More than half wanted to know what lifestyle changes to make to prevent a similar episode from occurring in the future.

I would really like to know what is ‘good’, what I should avoid and what can cause me trouble. *(Participant 4, 75–84 years, 3rd interview, RHF with COPD).*

Patients also wanted to know more about their prognosis and how to self-care.

I would like to know my progress and what the future will be like. I also want to know how I can take care of myself at home. In case something goes wrong I will not be afraid and will know what to do. *(Participant 7, 45–54 years, 1st interview, DCM).*

## Discussion

### Summary of findings

These findings reveal that patients’ illness experience is characterized by multidimensional distress and unmet information needs. Distressing physical symptoms such as acute breathlessness caused significant anxiety and were perceived as life-threatening requiring emergency care [13, 27, 28]. Conversely, symptoms with gradual onset were harder to recognize, and as they did not appear to interfere with daily life and were not seen as warranting immediate care. Indeed, the use of metaphorical local language to describe symptoms as ‘ordinary’ or ‘usual’ points to the way in which these symptoms were explained away and attributed to common acute illness and activities of daily life. Normalizing symptoms allowed patients to minimize the impact of their illness and continue with normal life activities such as returning to work to meet their financial needs. Although normalizing symptoms may have also helped patients to maintain a sense of routine life and continuity, normalizing prevented patients from the understanding that they were living with a life-threatening illness [29, 30].

The need to maintain a sense of continuity is also exemplified in patients’ expectation of a cure. The onset of chronic illness can significantly disrupt a usual sense of continuity which shifts from a predictable trajectory to one that is uncertain [31]. Patients whose symptoms were responsive to treatment felt hopeful that a cure was imminent. However, when acute exacerbations occurred patients struggled to make sense of an illness which increasingly became unfamiliar and lacked uniformity [32] with past illness history of acute treatable illness. In particular, younger patients expressed stronger feelings of anxiety and became increasingly frustrated with an illness preventing them from working to meet societal expectations to support the needs of their families [33]. At its core, preference for a HIV diagnosis shows that effective symptom control is important to patients. Poor symptom management leads to functional decline, further loss of independence and greater reliance on social networks for material support. The financial burden of accumulating health care and basic needs cost as well as needing to take time away from work may significantly reduce the capacity of family members to offer long term informal care [34]. From bereavement interviews we found that some patients received little help from their families as their condition deteriorated and as they became increasingly dependent. We have also described dwindling support from other traditional networks such as churches which patients perceive as being insensitive to their needs [26, 35]. Although we note that a few patients in formal employment benefitted from social health insurance, access to financial protection to guard against catastrophic costs of chronic illness is limited in Kenya. About 83% of people lack health insurance. However, plans are underway to expand coverage to include people who are informally employed who make up majority of the Kenyan workforce [36–38].

Our findings also reveal significant unmet information needs. Although most patients were aware they had a heart problem few patients understood what this meant. As an epidemiological shift from communicable to non-communicable disease is taking place in SSA patients understanding of heart failure remains low. Poor knowledge levels presents significant challenges for how patients recognize and understand symptoms as well as how they seek care [39]. Interestingly we found patients wanted to know how to prevent their illness signifying a belief, from their perspective, that their illness was acute and preventable. Our findings are similar to those of Namukwaya et al. [17] in Uganda who found that patients wanted to understand their illness, management and prognosis. Specifically, information was most needed at diagnosis and during acute crisis when symptoms became less familiar and were unresponsive to treatment. Similar information needs have been identified in studies conducted in the UK [40, 41]. Barriers which have been identified to limit effective communication and information exchange include poor prognostication of heart failure, patients fear of rebuke for making enquires, patients declining cognitive function which decreases their ability to remember which questions to ask and care mainly focused on achieving cure and overlooks psychological, social and spiritual needs which makes patients feel neglected and less likely to seek information [11, 14, 42].

### Implications for practice

Countries in SSA are currently facing an increasing burden of non-communicable diseases including heart failure [43, 44]. So far, epidemiological studies point to heart failure as a major public health issues with implications for development of appropriate services [8, 45]. What our findings show is that patients are struggling to cope and come to terms with this emerging illness. Using their own language and words Kenyan patients have described physical, social, spiritual, financial and information needs associated with their illness.

We propose that patients would benefit from a holistic approach providing multidimensional care. Palliative care is an appropriate holistic approach which has the potential to reduce suffering, support patients to live an active life as much as possible until death. In places where chronic disease services are in early stages of development, palliative care has the potential to also mitigate, through effective symptom management, the vicious cycle of poverty associated with chronic illness [46, 47]. Interventions aimed at improving communication with health professionals and supporting patients access to timely information are needed to enhance symptom management and patients’ quality of life [48]. Efforts to integrate palliative care should be reinforced by broader strategies to improve chronic disease management including promotion of primary prevention strategies, early disease detection, and prompt initiation of disease modifying therapy [2]. These should also include public health strategies aimed at education, health promotion and facilitating self-management [2]. Initiatives that strengthen health systems by improving health governance, information systems, resource mobilization, health financing and human workforce management should also be supported [49, 50].

### Strength and limitations

This is the first longitudinal qualitative study to explore the experiences of patients with heart failure in Kenya. A longitudinal study supported by the extended engagement in fieldwork allowed in depth exploration and generation of rich accounts of patients’ experiences [51] . This work also adds ethnic and geographical variation to longitudinal research on the experiences of living with heart failure which has, until recently, been conducted in mostly economically developed countries [16]. These findings broaden existing literature on heart failure in Sub Saharan Africa which has mainly focused on understanding an emerging disease epidemiology [4]. While we sought to recruit participants with varying sociodemographic, religious and cultural backgrounds our research was located in a specific geographical area. Caution is therefore needed when generalizing these findings to other regions in Kenya and Sub Saharan Africa. Finally, this was a small sample of 18 participants, but our experience in conducting in-depth serial interviews in other studies [52, 53], and our later interviews in this study suggest that we were approaching data saturation.

## Conclusions

People with heart failure in Kenya suffer significant physical, social, psychological, spiritual and financial distress. They also have considerable unmet information needs. Patients would benefit from a holistic approach to care, such as palliative care, which offers multidimensional care and aims to improve quality of life. Palliative care services should be provided alongside chronic disease management aimed at primary prevention of risk factors, early identification and initiation of disease modifying therapy [54]. Further research is needed to identify best practice for integrating chronic disease management and palliative care for patients with heart failure in Kenya and the greater SSA region.

## Abbreviations

COPD: Chronic obstructive pulmonary disease
DCM: Dilated cardiomyopathy
HHD: Hypertensive heart disease
HIV: Human immunodeficiency virus
RHF: Right sided heart failure
SSA: Sub Saharan Africa
